# Continuity and new perspectives at *Frontiers in Zoology*

**DOI:** 10.1186/s12983-026-00636-z

**Published:** 2026-10-09

**Authors:** Angelika Stollewerk

**Affiliations:** Queen Mary University, London, UK

Changes in editorial leadership are moments to look back, to say thank you, and to look ahead. After twelve years as Co-Editor-in-Chief of *Frontiers in Zoology*, Ulrich Technau stepped down from this role in July 2026. Uli joined the journal as Co-Editor-in-Chief in 2014, succeeding Diethard Tautz, and subsequently led the journal together with Jürgen Heinze for more than a decade. Following Jürgen’s departure at the end of 2024, I had the pleasure of working alongside Uli as Co-Editor-in-Chief.

During his twelve years in this role, Uli made an important contribution to maintaining the scientific quality and broad zoological scope of *Frontiers in Zoology*. Zoology encompasses an extraordinary diversity of organisms, disciplines and approaches, and maintaining a journal that reflects this breadth requires both scientific expertise and an openness to research extending well beyond one’s own field. Uli brought both qualities to the journal. His own research addresses fundamental questions concerning the origin and evolution of animal body plans, using cnidarians as models to investigate the genomic, molecular and developmental basis of key features of animal organization, including germ layers and nervous systems. His expertise in molecular evolution and evolutionary developmental biology has therefore been particularly valuable to a journal dedicated to integrating the many different disciplines of modern zoology.

Beyond his scientific expertise, Uli has been deeply committed to the journal itself. Maintaining the quality of a scientific journal depends not only on selecting and evaluating individual manuscripts, but also on building and developing an Editorial Board whose collective expertise reflects the full breadth of the field. Uli contributed greatly to identifying and recruiting Associate Editors from different areas of zoology and from the international scientific community. The close exchange between the Editors-in-Chief and Associate Editors provides an important network of expertise that helps shape the scientific profile of the journal, while at the same time connecting *Frontiers in Zoology* to the wider zoological community. Uli fostered this exchange throughout his time as Co-Editor-in-Chief, while continuously promoting the journal and considering how it could develop in response to changes in zoological research and scientific publishing.

On a personal level, I am particularly grateful for the excellent collaboration we have had since I joined him as Co-Editor-in-Chief in January 2025. His experience, thoughtful judgement and willingness to discuss both individual manuscripts and broader editorial questions made my transition into this role at *Frontiers in Zoology* considerably easier and made working together a genuine pleasure.

On behalf of *Frontiers in Zoology*, its Editorial Board and the German Zoological Society (DZG), I would therefore like to thank Uli most warmly for his many years of commitment to the journal and for everything he has contributed to its development.

At the same time, we are very pleased to welcome Stefan Richter as the new Co-Editor-in-Chief of *Frontiers in Zoology*. Stefan is Professor of General and Special Zoology at the University of Rostock. His research spans morphology, systematics, phylogenetics and evolutionary biology, with a particular focus on understanding organismal diversity and evolutionary relationships. He also brings extensive editorial experience from a number of journals in zoology, systematics and evolutionary biology.

Stefan has a particularly strong connection to the DZG. He served on the Executive Board of the Society for many years and was DZG President in 2017–2018. His appointment as Co-Editor-in-Chief therefore comes at an especially appropriate time, as *Frontiers in Zoology* is strengthening the integration between the journal and its founding society.

As part of this development, the journal is currently introducing a new editorial structure designed to make the breadth of zoological research represented in *Frontiers in Zoology* more visible. The expertise of the Editorial Board is being organised into the major subdisciplines of zoology, and authors will be able to submit their work directly to the section most appropriate for their research. Members of the DZG’s corresponding subdiscipline groups have joined the journal as Section Editors and help develop these sections, further strengthening the connection between the international Editorial Board and the scientific community represented by the DZG.

This new structure reflects an important principle that has guided *Frontiers in Zoology* since its foundation: zoology gains its strength from the diversity of its disciplines and from bringing together different approaches to understanding animals, from molecules, development and physiology to morphology, behaviour, ecology, evolution, systematics and biodiversity. The new sections are intended to make this breadth more visible while maintaining the high standards of scientific quality that remain at the heart of the journal.

We are delighted to welcome Stefan to *Frontiers in Zoology* and look forward to working together with him, our Editorial Board, the DZG and our authors to further develop the journal and promote excellent research across the full breadth of zoology. At the same time, we once again extend our warmest thanks to Uli for twelve years of dedicated service and wish him all the very best for the future.

Angelika Stollewerk.

